# Cross-level Cross-Scale Inference and Imputation of Single-cell Spatial Proteomics

**DOI:** 10.21203/rs.3.rs-7108570/v1

**Published:** 2025-07-28

**Authors:** You Wu, Lei Xie

**Affiliations:** 1 Ph.D. Program in Computer Science, The Graduate Center, The City University of New York, New York, New York, USA; 2 School of Pharmacy and Pharmaceutical Sciences & Center for Drug Discovery, Northeastern University, Boston, MA, USA; 3 Helen & Robert Appel Alzheimer’s Disease Research Institute, Feil Family Brain & Mind Research Institute, Weill Cornell Medicine, Cornell University, New York, New York, USA

## Abstract

High-throughput single-cell and spatial omics technologies have transformed biological research. Despite these advances, reliably identifying the molecular drivers and their interplays across biological levels and scales remains a significant challenge. Current experimental methods are limited by batch effects, the lack of simultaneous multi-modal measurements in individual cells, limited coverage of measured proteins, poor generalization to unseen conditions, and insufficient spatial context at a single-cell resolution. To overcome these challenges, we introduce scProSpatial, a unified, multi-modal, multi-scale deep learning framework designed to infer and impute high fidelity single-cell spatial proteomics from scRNA-seqs. Through comprehensive evaluations, scProSpatial accurately predicts spatial abundances of proteins in the absence of shared transcriptomics features, expands protein coverages by 50 times, and generalizes robustly to out-of-distribution scenarios. A case study in metastatic breast cancer further illustrates its utility, demonstrating scProSpatial’s potential to drive cross-level, cross-scale multi-omics integration and analysis and reveal deeper insights into complex biological systems.

## 1 Introduction

Single-cell and spatial omics profiling technologies have transformed our ability to dissect cellular heterogeneity and spatial organization. However, major challenges remain in bridging data across modalities, particularly between transcriptomic and proteomic measurements. Current single-cell multi-omics methods such as CITE-seq (Cellular Indexing of Transcriptomes and Epitopes by sequencing) enable jointly profiling RNAs and surface proteins in suspension [1, 2], yet they lack spatial context and only covers typically hundreds of proteins. Conversely, spatial proteomics platforms like CODEX can localize dozens of proteins in situ, but at very low protein coverage (typically 30–60 markers) and lack a single-cell resolution. As a result, there is currently few assay capable of simultaneously measuring the transcriptome and proteome at high spatial resolution within the same cell and with a high protein coverage. This gap poses two fundamental challenges for data integration: (1) the absence of spatially resolved, matched RNA–protein measurements and (2) the low multiplexity and resolution of existing spatial proteomics platforms.

An intermediate layer of paired information may bridge these gaps. Although CITE-seq provides matched RNA and surface protein measurements, it is constrained to surface markers and does not capture intracellular proteomes. Spatial proteomic data, on the other hand, are often too sparse and variable across tissue samples to support accurate mapping from transcript-derived predictions. Batch effects and low protein coverage further hinder robust cross-modal integration, emphasizing the need for new strategies to unify transcriptomic and proteomic information in a single cell resolution and in a spatially resolved manner.

While machine learning approaches have emerged to predict surface protein abundance from transcriptomic data, such as TotalVI[3], sciPENN [4], and scTranslator[5], these methods face significant limitations. They often struggle to generalize to unseen conditions and do not integrate effectively with spatial omics data. Maxfuse[6] stood out as one of the first models to match omics data to the spatial domain, but it requires pre-defined linked features to initialize the model.

To overcome these challenges, we developed scProSpatial, a multi-scale deep learning platform that explicitly models the gap between single-cell transcriptomics and spatial proteomics. scProSpatial’s core architecture consists of three stages: (1) a pre-training phase in which a Transformer-based large language model is trained on large, heterogeneous RNA and protein with masked token prediction and PPI-guided encoding to learn context-aware representations; (2) a fine-tuning phase in which the pre-trained encoders are aligned using paired CITE-seq data via a contrastive loss, enabling accurate RNA-to-surface-protein translation; and (3) downstream task that predicts missing surface protein abundances from single-cell RNA, and maps those predictions onto spatial proteomics images, without requiring any shared marker panel. scProSpatial achieves robust domain alignment even when spatial assays measure only a limited set of proteins at coarse resolution. We demonstrate scProSpatial’s ability to predict thousands of spatially localize proteins at single-cell resolution at a zero-shot setting. In addition, we present its use in metastatic breast cancer at multiple sites. This linked-feature-free framework enables high-resolution integration of transcriptomics and proteomics, paving the way for more comprehensive phenotypic and spatial analyses of complex organisms.

## 2 Results

### 2.1 Overview of scProSpatial

scProSpatial employs a two-stage training strategy designed to effectively integrate multi-modal and multi-scale omics data. The first stage involves self-supervised pre-training to learn robust, domain-aware representations from diverse biological datasets. The second stage utilizes contrastive learning to align representations from different modalities, specifically RNA and protein expressions, into a shared embedding space. The model’s capabilities are subsequently evaluated through comprehensive benchmark studies using paired multi-modal data and zero-shot case studies involving unpaired single-cell and spatial data 1.

In the initial pre-training stage, scProSpatial integrates multiple datasets by self-supervised training. Input datasets include paired multi-modal data (three CITE-seq datasets: liver tissue, PBMC, BMMC, see Method4) and datasets for case studies featuring unpaired single-cell RNA-seqs and spatial proteomics (CODEX technology for human tonsil, metastatic breast cancer tissues). The core architecture consists of two parallel encoders, one for RNA expression and one for protein abundance. Pre-training employs a masked token prediction objective, akin to masked language modeling. Crucially, this stage incorporates protein-protein interaction (PPI) network information to guide the representation learning, potentially serving as a biologically informed inductive bias. To integrate data from diverse sources and batches effectively, we employ learnable batch tokens and padding strategies, as used in scGPT [7]. This self-supervised phase aims to build foundation encoders that understand the intrinsic patterns within transcriptomic and proteomic data.

Following pre-training, the second stage focuses on aligning the RNA and protein modalities. Using paired datasets (specifically, the CITE-seq data where RNA and surface protein measurements are available for the same cells), the pre-trained encoders are fine-tuned. This fine-tuning leverages a contrastive loss function, inspired by CLIP[8]. The objective is to map corresponding RNA and protein profiles from the same cell close together in a joint embedding space, while pushing representations from different cells apart. This step is critical for enabling cross-modal tasks, such as predicting protein abundance from RNA expression. The impact and optimization of this contrastive alignment stage are further analyzed in the following sections.

The model evaluation is performed through two main approaches. First, we conduct a benchmark study using paired CITE-seq datasets to rigorously test RNA-to-protein translation accuracy, assessing performance under random split, few-shot, and few-shot with out-of-distribution (OOD) conditions. To demonstrate utility in challenging real-world scenarios lacking paired data, we then perform zero-shot case studies using human tonsil and metastatic breast cancer datasets. In these studies, scProSpatial predicts cell-level protein profiles from scRNA-seq and subsequently maps these predictions onto spatial spot-level CODEX data through the learned joint embeddings and a similarity-based matching algorithm. Detailed illustration is provided in Supplemental Figure S1 and Method4.

### 2.2 scProSpatial Reliably Translates Transcriptomics to Proteomics

**Figure 2:**
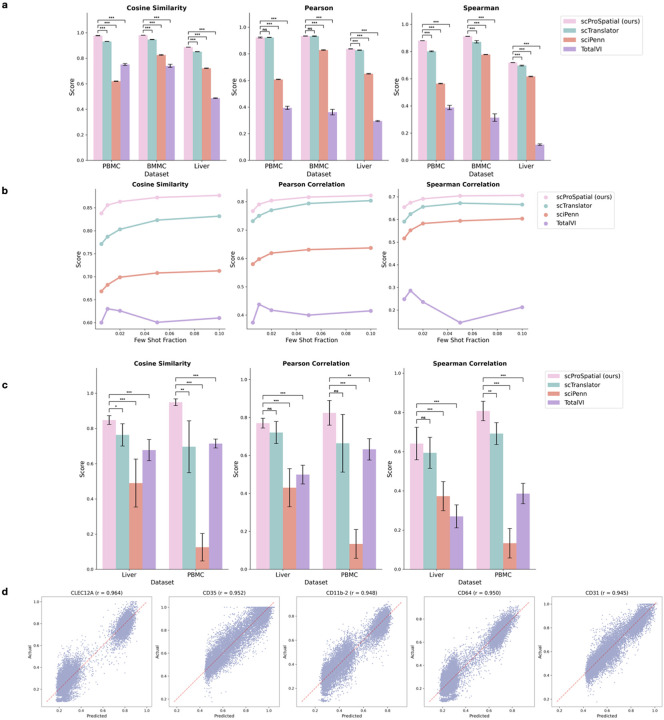
scProSpatial performance compared to baseline models in predicting surface protein abundance from single-cell transcriptomics. a, Standard random split evaluation. b, Few-shot learning with training on small cell fractions (10%, 5%, 2%, 1%, and 0.5%). c, Few-shot combined with out-of-distribution (OOD) generalization: models trained on a small subset and tested on held-out cell types. d, Scatter plots for five marker proteins on the PBMC test set. Error bars represent five independent runs (a: random seeds; c: different shots). Significance: ***p<0.001,**p<0.01,*p<0.05, ns = not significant.

The major task of scProSpatial is to translate transcriptomics to the corresponding protein abundance at single-cell and spatial resolutions. We performed experiments using the PBMC, BMMC, and liver CITE-seq datasets, which provide paired measurements of RNA and surface proteins within the same cells, allowing for direct evaluation of translation accuracy. We assessed scProSpatial’s performance against a series of baseline methods, including TotalVI[3], sciPenn[4] and scTranslator[5], and under three different evaluation scenarios, as shown in Figure 2.

In the standard supervised setting (Figure2a), scProSpatial significantly outperformed all baselines across all datasets. It achieved the highest cosine similarity (0.95), Pearson (0.90), and Spearman (0.84) correlations, surpassing scTranslator, sciPenn, and TotalVI by wide margins. Paired t-tests confirmed the improvements were statistically significant (p<0.001) in nearly all comparisons, particularly for cosine and Spearman metrics. These results highlight scProSpatial’s strong predictive accuracy and robust generalization when ample paired training data are available.

To evaluate performance under data scarcity, we simulated a few-shot learning scenario on the liver dataset by training with only small fractions of cells (10%, 5%, 2%, 1%, and 0.5%). Figure 2b shows that scProSpatial maintained strong performance even at the lowest fractions: at 0.5% of training cells, scProSpatial’s cosine similarity remained above 0.85 and rose to over 0.88, whereas scTranslator started around 0.78 and only reached about 0.82, sciPenn stayed around 0.65–0.70, and TotalVI remained below 0.65. These results demonstrate scProSpatial’s robustness to very limited cell counts.

In the most challenging few-shot combined with out-of-distribution (OOD) scenario, scProSpatial was trained on a small subset of cells and tested on completely different held-out cell types (Figure2c). It achieved the highest performance across all metrics, with cosine similarity (0.90), Pearson (0.80), and Spearman (0.72) scores, significantly outperforming sciPenn and TotalVI (p<0.001) and showing consistent gains over scTranslator (p<0.05 for most comparisons). While Pearson and Spearman differences with scTranslator were not significant on liver data, scProSpatial maintained strong performance across datasets, demonstrating superior generalization to unseen cell types under limited supervision.

Finally, Figure 2d presents scatter plots for five marker proteins on the PBMC test set. Each panel plots predicted versus actual abundance across all test cells, with a diagonal line indicating perfect agreement. scProSpatial’s predictions cluster tightly around the diagonal, yielding Pearson correlation values of the average of 0.95. This visualization confirms that scProSpatial not only achieves high aggregate metrics but also captures fine-grained expression patterns for individual proteins.

### 2.3 scProSpatial Effectively Aligns Two Domains Through Contrastive Learning

A key component of scProSpatial is the contrastive learning stage, designed to align transcriptomic and proteomic representations into a shared embedding space. To evaluate the effectiveness of this alignment, we conducted several experiments assessing cross-domain transfer, matching task improvement, and direct alignment quality.

**Figure 3:**
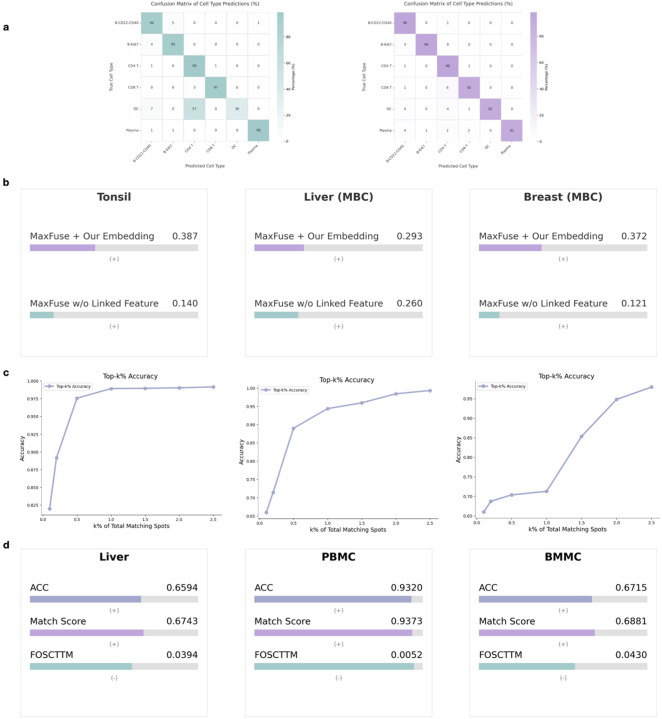
Alignment analysis. a, Confusion matrices show true vs. predicted cell types of the human tonsil dataset using RNA and protein embeddings aligned via contrastive learning, with encoders trained only on PBMC CITE-seq. b, comparison with Maxfuse on the matching task when the pre-defined linked features are unavailable. Note: *MaxFuse+Embedding* is MaxFuse using our embeddings as the linked features. c, top-k accuracy on the”M cell-type matching task on the unpaired datasets. d, a direct quantitative evaluation of the alignment quality on held-out data from the paired CITE-seq datasets. Note: MBC: Metastatic breast cancer

We first tested whether the learned representations could generalize across datasets and aid cell type annotation. Using the encoders aligned via contrastive learning that were trained solely on the PBMC dataset, we created a cell type prediction task with the unpaired human tonsil dataset (Methods4). Figure 3a presents the confusion matrices comparing the true cell type annotations against predictions derived from the aligned embeddings, using both RNA and protein encoders, shown separately. The strong diagonal patterns in both matrices show high prediction accuracy across most cell types. This demonstrates that scProSpatial successfully learns biologically meaningful features that are aligned across modalities from RNAs to proteins and tissue contexts from PBMC to tonsil, validating the quality of the domain alignment.

Spatial integration methods often rely on linked features, especially when the paired information is unavailable. MaxFuse [6] is one example, but its performance can degrade without such linked features. We investigated whether scProSpatial’s learned joint embeddings could serve as a substitute initialization to improve MaxFuse’s performance in unpaired scenarios. We applied MaxFuse to map scRNA-seq data to spatial proteomics data for the human tonsil dataset, metastatic breast cancer liver site, and breast site (liver and breast MBC). We compared the mapping performance using MaxFuse initialized with scProSpatial’s embeddings versus a baseline MaxFuse without linked features. As shown in Figure 3b, incorporating scProSpatial’s embeddings resulted in substantially improved alignment scores across all three datasets. This highlights the practical utility of contrastively learned embeddings by scProSpatial for improving downstream spatial data integration, particularly when a prior knowledge on linked marker genes is lacking.

To directly assess whether the joint embedding space captures biological similarity across modalities, we calculated the Top-k% cell type matching accuracy on the human tonsil and metastatic breast cancer dataset. This metric evaluates, for a given cell’s transcriptomic profile, the probability that its correct cell type label is represented among the top k% most similar entities (cells or spots) in the spatial proteomics data, based on proximity in the learned embedding space. The curves in Figure 3c demonstrate that the Top-k% accuracy rapidly increases as k grows, reaching high levels (e.g., >80–90%) even for small percentages of k. This confirms that the contrastive alignment effectively positions corresponding biological entities from the transcriptomic and proteomic domains close to each other within the shared embedding space.

Finally, we performed a direct quantitative evaluation of the alignment quality on held-out data from the CITE-seq datasets used during fine-tuning. We employed standard cross-modal retrieval metrics: Accuracy (ACC), Match Score, and Fraction of Samples Closer Than True Match (FOSCTTM). As shown in Figure 3d, scProSpatial achieved high ACC and Match Scores and critically, very low FOSCTTM values. These results provide direct quantitative evidence that the contrastive learning stage successfully aligns the paired multi-modal data.

### 2.4 Zero-Shot Spatial Proteome Prediction Improves Understanding of Liver Metastasis of Breast Cancer

To demonstrate scProSpatial’s utility in a complex, real-world scenario lacking paired multi-omics data, we performed a zero-shot case study on the metastatic breast cancer (MBC) dataset profiling liver tissue using unpaired scRNA-seq and CODEX spatial proteomics. scProSpatial was employed to predict spatial protein expression patterns directly from the scRNA-seq data after mapping cells to the spatial coordinates defined by CODEX imaging.

**Figure 4:**
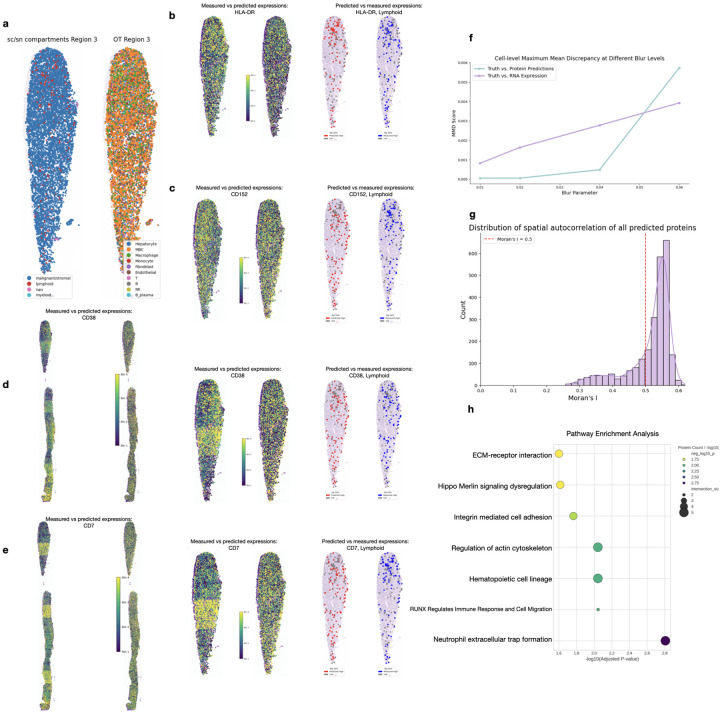
Evaluation of predicted protein expression in liver tissue. a, Spatial distribution of major cell compartments and cell types from scRNA-seq annotations. b–e, Comparison of predicted vs. measured expression for representative proteins across the full tissue (left, four expression bins) and within relevant compartments (right, high predicted in red, high measured in blue). f, Spatial concordance assessed via cell-level Maximum Mean Discrepancy (MMD) between predictions and ground truth, with RNA expression as a baseline. g, Distribution of Moran’s I scores showing spatial autocorrelation across all predicted proteins. h, Pathway enrichment analysis of the most spatially informative predicted proteins.

First, we established the spatial context of the tumor microenvironment (TME). Figure 4a illustrates the spatial distribution of major cell compartments (malignant/stromal, lymphoid, myeloid) derived from scRNA-seq annotations, alongside the locations of detailed cell types (including Hepatocytes, MBC cells, Macrophages, T cells, B cells, etc.) within a representative tissue region[9]. We then focused on evaluating the predicted spatial expression patterns against the measured CODEX data for key protein markers relevant to the TME (Figure 4b–e). We visualized patterns for HLA-DR, an antigen presentation marker indicating immune activity, CD152/CTLA-4, an immune checkpoint marker, CD38–an activation marker, and CD7, a T-cell/NK cell marker.

In general, the predicted spatial protein distributions in red dots demonstrated good agreement with the measured CODEX signals, correctly localizing markers to their expected compartments within the TME. For each protein, the left panels visualize full-region expression patterns using binned intensity levels (Bin 1–4), the right panels offer a more focused view of expression within the certain compartment, with high-expression regions using binarized overlays. Specifically, HLA-DR (Figure 4b) showed widespread but patchy distribution in the full-region view measured data. scProSpatial’s prediction captured the same widespread localization but presented a smoother pattern, potentially offering a clearer view of regions with high immune activity. The binarized plots show denser predicted (red) signal within the high-expression zones compared to the measurement (blue). CD152/CTLA-4 (Figure 4c)’s measured expression forms moderate, spatially clustered patterns, scProSpatial effectively captured these clusters, especially within lymphoid compartments. In the very left of Figure 4d, the measured signal for this activation marker in the full-region view appeared very patchy, suggesting artifacts, predictions showed more continuous and structured patterns, potentially denoising the signal while preserving compartment specificity. Likewise, the measured signal for this T/NK cell marker(Figure 4e) was notably sparse and patchy. In contrast, scProSpatial’s prediction, while localized to the same general regions, showed much denser and more complete patterns. This strongly suggests that the prediction can effectively “fill in” biologically expected patterns, potentially correcting for technical artifacts like dropout or variable staining efficiency in the experimental measurements.

We quantitatively assessed the spatial concordance using Cell-level Maximum Mean Discrepancy (MMD), comparing spatial distributions derived from ground truth annotations, predicted proteins, and input RNA across different spatial scales (blur parameters, Figure 4f). At finer scales, the predicted protein distribution matched the ground truth more closely than the input RNA distribution did. However, this trend reversed at the coarse scale, where the RNA-derived distribution showed a lower MMD than the protein predictions. This suggests that scProSpatial may improve the representation of local spatial organization, though the effect varies depending on the scale of analysis.

Furthermore, we assessed the spatial structure inherent in the entire predicted proteome, which included 2435 proteins not measured by CODEX in addition to the 49 measured ones (2484 total). We calculated the spatial autocorrelation –Moran’s I score for each predicted protein. The distribution shown in Figure 4g reveals strong positive spatial autocorrelation for the vast majority of proteins, with most Moran’s I values exceeding 0.5. This confirms that scProSpatial generates spatially coherent and biologically plausible expression patterns across the tissue, even for proteins absent in the experimental panel.

Finally, we explored the biological processes associated with the most spatially informative proteins predicted by scProSpatial (Figure 4h). We performed pathway enrichment analysis using the top 20 predicted proteins that exhibited the highest autocorrelation scores (Moran’s I score), revealing critical pathways highly relevant to breast cancer progression and metastasis. ECM-receptor interaction pathways emerged as the most significantly enriched, consistent with the well-established role of extracellular matrix remodeling in tumor progression and metastasis [10, 11, 12]. Regulation of actin cytoskeleton was prominently featured, reflecting its fundamental role in driving epithelial-mesenchymal transition (EMT), cell migration, and invasive potential in breast cancer [13, 14]. Hippo signaling dysregulation, characterized by YAP/TAZ hyperactivation, was also identified, which is implicated in breast cancer proliferation, stemness, and resistance to therapy [15, 16]. Integrin-mediated cell adhesion pathways were enriched, highlighting their essential roles in cell-ECM interactions, migration, angiogenesis, and the metastatic cascade in breast cancer [17, 18]. RUNX3-regulated pathways appeared in our analysis, consistent with its role as a tumor suppressor frequently downregulated in breast cancer and involved in TGF- signaling [19, 20]. Hematopoietic cell lineage pathways likely reflect the complex tumor microenvironment and immune cell infiltration patterns characteristic of breast cancer tissues [21]. Neutrophil extracellular trap formation was also detected, which has recently been linked to cancer metastasis and poor prognosis. These pathway enrichments collectively underscore how spatially informative proteins identified by scProSpatial capture key biological processes driving breast cancer pathogenesis, providing mechanistic insights into the spatial organization of tumor biology.

### 2.5 Zero-shot scProSpatial Characterizes the Primary Breast Tumor Microenvironment

Complementing the analysis of the liver site of the metastatic breast cancer tissue, we applied scProSpatial in a zero-shot manner within the breast site.

**Figure 5:**
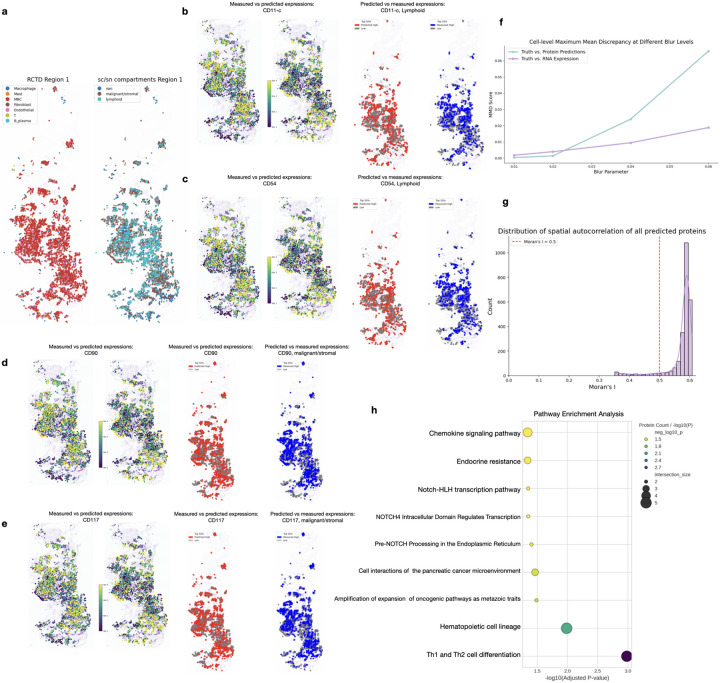
Evaluation of predicted protein expression in breast tissue. a, Spatial distribution of major cell compartments and cell types from scRNA-seq annotations. b–e, Comparison of predicted vs. measured expression for representative proteins across the full tissue (left, four expression bins) and within relevant compartments (right, high predicted in red, high measured in blue). f, Spatial concordance assessed via cell-level Maximum Mean Discrepancy (MMD) between predictions and ground truth, with RNA expression as a baseline. g, Distribution of Moran’s I scores showing spatial autocorrelation across all predicted proteins. h, Pathway enrichment analysis of the most spatially informative predicted proteins.

Figure 5a illustrates the spatial distribution of major cell compartments (malignant/stromal, lymphoid) derived from scRNA- seq annotations, alongside the locations of detailed cell types (including MBC cells, fibroblasts, macrophages, T cells, and plasma cells mapped using RCTD [9]). We then focused on evaluating the predicted spatial expression patterns against the measured CODEX data for key protein markers relevant to the TME (Figure 4b–e). Consistent with observations in the liver tissue, predicted patterns generally showed good spatial agreement with measurements, often appearing smoother or complementary effects.

For instance, predicted CD11c (Figure 5b, dendritic cell/monocyte marker) and CD54 (ICAM-1, Figure 5c, adhesion/inflammatory marker) correctly localized to lymphoid-rich or interface regions. Predicted CD90 (Thy-1, Figure 5d, stromal/stem cell marker) was enriched in malignant/stromal compartments. Predicted CD117 (c-Kit, Figure 5e, receptor tyrosine kinase) patterns closely matched the measured signal, potentially highlighting specific tumor cell clones or mast cells. In several cases (e.g., CD11c, CD90), the predictions (red dots) provided a more contiguous view compared to the sparser measurements (blue dots).

Quantitative analysis of cell-level spatial distributions using MMD (Figure 5f) showed a similar scale-dependent trend as observed in the liver tissue: protein predictions provided a close match to the ground truth organization at finer spatial scales, while the RNA-derived distribution aligned better at the coarser scale.

Furthermore, spatial autocorrelation analysis (Figure 5g) confirmed that nearly all predicted proteins (including measured and unmeasured) exhibited strong positive spatial structure (Moran’s I peaking near 0.58–0.60), suggesting of biologically realistic organization within the primary tumor TME even at zero-shot scnerio.

Following the same approach, we used the top predictive proteins for pathway enrichment analysis as shown in Figure 5h, revealing several pathways strongly associated with breast cancer biology and clinical outcomes. Th1 and Th2 cell differentiation emerged as a significant pathway, reflecting critical immune cell polarization dynamics in the tumor microenvironment where [22, 23] Th1/Th2 imbalance contributes to immune evasion and therapeutic resistance in breast cancer. Chemokine signaling pathways, particularly the CXCL12/CXCR4 axis, were prominently enriched and represent [24, 25] master regulators of breast cancer tumorigenesis, controlling tumor cell proliferation, motility, and metastasis to target organs while affecting both immune and stromal cells to create tumor-supporting microenvironments. Endocrine resistance pathways were identified, reflecting [26, 27] the critical clinical challenge in ER-positive breast cancer where resistance to tamoxifen and aromatase inhibitors develops through complex molecular mechanisms involving growth factor signaling crosstalk and epigenetic modifications. NOTCH signaling pathways (NOTCH4, Notch-HLH, Pre-NOTCH processing) were significantly enriched, consistent with [28, 29] NOTCH1 and NOTCH4 promoting stemness, therapy resistance, and aggressive phenotypes particularly in basal-like and triple-negative breast cancers. Additionally, amplification and expansion of oncogenic pathways as metastatic traits were detected, likely reflecting pathway enrichment of known metastatic drivers and oncogene activation signatures. These pathway enrichments collectively demonstrate how spatially informative proteins identified by scProSpatial capture key biological processes governing primary tumor biology and its potential for metastatic progression.

## 3 Discussion

Resolving the spatial distribution of proteins within tissues remains a fundamental challenge in systems biology. While single-cell and spatial omics technologies offer complementary insights, the lack of integrated, high-resolution measurements across modalities has limited our ability to reconstruct tissue-level protein architecture with molecular specificity. scProSpatial addresses this gap by establishing a cross-level, cross-scale framework that translates single-cell transcriptomic data into single-cell spatial proteomic representations without requiring matched features or paired training data.

Through extensive benchmarking on CITE-seq datasets, scProSpatial consistently achieved higher predictive accuracy than existing methods under standard, few-shot, and out-of-distribution conditions. Its resilience to limited training data and capacity to generalize across cell types suggests a level of robustness that is critical for practical applications, particularly in disease contexts where sample constraints are common.

The contrastive learning framework aligns RNA and protein modalities into a shared embedding space that captures biological similarity. This structure enables zero-shot transfer of transcriptomic profiles to spatial proteomic domains. Evaluations on unpaired datasets, including tonsil and metastatic breast cancer tissues, confirm the model’s ability to reconstruct tissue architecture and marker localization at single-cell-level resolution.

In metastatic breast cancer tissues, the inferred protein maps reconstructed tissue compartment boundaries, recovered spatial gradients for key markers, and yielded autocorrelated expression patterns even for proteins not present in the experimental panel. Pathway enrichment analyses of the top spatially structured proteins uncovered signaling axes consistent with the known pathophysiology of breast cancer, including ECM remodeling, actin dynamics, and immune cell trafficking. These results suggest that the predicted proteomic landscape is not only spatially coherent but also biologically informative.

Several limitations remain. The current model focuses on surface proteins and does not account for intracellular or post-translationally modified proteins. Its spatial projection is based on embedding similarity and does not incorporate histology or morphology. Future work may incorporate image-guided refinement.

scProSpatial enables cross-level, cross-scale inference in biological systems where direct measurement is limited. As spatial and single-cell technologies continue to advance, such frameworks will be critical for linking molecular-level to tissue-scale organization and generating new insights into complex tissues or incurable diseases.

## 4 Methods

scProSpatial integrates single-cell transcriptomics and spatial proteomics through a two-stage training strategy (Supplemental Figure S1). First, two parallel transformer-like encoders are pretrained on a mix of datasets using a masked language modeling (MLM) objective to learn domain-aware embeddings. Second, RNA and protein modalities are aligned via contrastive learning. We evaluate the model on paired CITE-seq data as a machine translation task (translating RNA to protein), followed by a case study applying zero-shot spatial mapping onto CODEX data from metastatic breast cancer tissues.

### 4.1 Pretraining

We assemble six datasets: three paired CITE-seq assays (liver, PBMC, BMMC) and three unpaired scRNA-seq assays (human tonsil, and two biopsy samples from metastatic breast cancer in liver and breast tissue). In each mini-batch, 30% of gene (or protein) features are masked (indices ℳ), and masked entries are replaced by a learnable token.

#### Gene embedding construction

For each token i, we compute an initial embedding by combining expression and PPI information:

$$g_{i}=x_{i}^{\text{expr}}+x_{i}^{\text{ppi}}$$

where $x_{i}^{\text{expr}}$ is the linearly projected expression value, $x_{i}^{\text{ppi}}$ is the precomputed PPI embedding.

#### Performer encoding

Stack gene embeddings into $G\in R^{n\times d}$:

$$G=[{\text{g}}_{1};\ldots ;{\text{g}}_{n}]$$

The Performer encoder processes G:

$$H=\text{Performer}(G)\;\in R^{n\times d},$$

producing contextualized token embeddings $h_{i}=H_{i}$.

#### Cell and gene representations

We derive:

$$\text{Gene}\;\text{embeddings}:\;h_{i}=H_{i},$$

$$\text{Cell}\;\text{embedding}:\;\text{c}=\frac{1}{n}\sum_{i=1}^{n}\;h_{i}.$$

#### Batch token integration

To account for dataset-specific variation, we assign each dataset d∈{1,…,D} a learnable embedding vector $b_{d}\in R^{d}$. After computing the cell embedding c as the average of contextualized gene embeddings $h_{i}$, we add the dataset embedding to obtain the final cell representation:

$$z=c+\alpha \cdot b_{d},$$

where α∈R is a learnable scaling parameter. This allows the model to encode dataset-level context into each cell representation.

#### Pretraining loss

A reconstruction head predicts masked entries directly from z, yielding predictions $\hat{x}[ℳ]$. The pretraining objective is:

$${ℒ}_{\text{pre}}=E_{ℳ}\|x[ℳ]-\hat{x}[ℳ]{\|}_{2}^{2}.$$

### 4.2 Ablation Study

We first performed a modular ablation study to assess the components contributing to effective masked language model (MLM) pretraining (Supplementary Table S1). We evaluated the use of dataset-specific tokens, PPI integration, and gene features. The largest improvement (34.4% loss reduction) came from adding learnable dataset tokens, followed by consistent gains from PPI integration and increasing input gene features from HVG to full genes. The combination of all three yielded the lowest MLM loss, and this configuration was used for downstream experiments.

We then tested the downstream utility of this optimized pretraining in few-shot learning settings (shot sizes: 0.1% to 0.005%) across three datasets (Supplementary Table S2), which simulate practical scenarios of limited supervision. This focus reflects our hypothesis that pretraining is most impactful when training data is scarce, where generalization benefits are expected to be amplified. Consistent improvements were observed across all metrics, with up to +8.3% Pearson and +6.3% Spearman correlation gain. While statistical significance was not reached (p>0.05), the consistent performance gains validate the practical benefit of pretraining, particularly in low-data regimes.

### 4.3 Contrastive Learning

To align RNA and protein embeddings in a shared latent space, we adopt a CLIP-style contrastive framework. Given a batch of N paired cells, let $e_{\text{rna}}^{i}=H_{0,i}^{\left(R\right)}$ and $e_{\text{prot}}^{j}=H_{0,j}^{\left(P\right)}$. Compute cosine similarity:

$$s_{i,j}=\frac{e_{\text{rna}}^{i}⊤e_{\text{prot}}^{j}}{\left\|e_{\text{rna}}^{i}\right\|\left\|e_{\text{prot}}^{j}\right\|}.$$

The RNA-to-protein loss is

$${ℒ}_{\text{rna}-\text{prot}}=-\frac{1}{N}\sum_{i=1}^{N}\text{log}\frac{\text{exp}\left(s_{i,i}/\tau \right)}{\sum_{j=1}^{N}\text{exp}\left(s_{i,j}/\tau \right)},$$

and the protein-to-RNA loss is

$${ℒ}_{\text{prot}-\text{rna}}=-\frac{1}{N}\sum_{i=1}^{N}\text{log}\frac{\text{exp}\left(s_{i,i}/\tau \right)}{\sum_{j=1}^{N}\text{exp}\left(s_{j,i}/\tau \right)}.$$

The final contrastive loss is

$${ℒ}_{\text{contrastive}}=\frac{1}{2}\left({ℒ}_{\text{rna}-\text{prot}}+{ℒ}_{\text{prot}-\text{rna}}\right).$$

### 4.4 Translational Prediction of RNA to Protein

We frame RNA→protein prediction as a machine-translation task. First, the RNA input $x_{\text{rna}}$ is encoded:

$$e_{\text{rna}}=E_{\text{rna}}\left(x_{\text{rna}}\right).$$

A translation MLP produces:

$$e_{\text{trans}}=\text{ML}{\text{P}}_{\text{trans}}\left(e_{\text{rna}}\right).$$

For each protein p, we query the protein encoder by its ID $p_{\text{id}}$, obtaining:

$$u_{p}=E_{\text{prot}}\left[b_{d};\;p_{\text{id}}\right].$$

We then compute a cross-attention between $u_{p}$ (as query) and $e_{\text{trans}}$ (as keys/values):

$$\alpha_{p}=\text{softmax}\left(\frac{u_{p}^{⊤}e_{\text{trans}}}{\sqrt{d}}\right),\;c_{p}=\alpha_{p}\;e_{\text{trans}},$$

and predict abundance via a linear head:

$${\overset{ˆ}{y}}_{\text{prot},p}=w_{o}^{⊤}\;c_{p}$$

### 4.5 Zero-Shot Spatial Matching

To map unpaired scRNA cells to CODEX spots, we compute embeddings {$z_{i}^{\text{rna}}$}, {$z_{j}^{\text{prot}}$} and similarity scores

$$S_{\text{ij}}=\frac{z_{i}^{\text{rna}}⊤z_{j}^{\text{prot}}}{\left\|z_{i}^{\text{rna}}\right\|\left\|z_{j}^{\text{prot}}\right\|}.$$

We derive soft assignments

$$W_{\text{ij}}=\frac{\text{exp}\left(S_{\text{ij}}/\tau_{m}\right)}{\sum_{k}\text{exp}\left(S_{\text{ik}}/\tau_{m}\right)},$$

and predict spatial protein expression at spot j:

$${\hat{x}}_{j}^{\text{prot}}=\sum_{i}W_{\text{ij}}{\overset{ˆ}{y}}_{\text{prot},p(i)},$$

where p(i) indexes the protein predicted for cell i.

### 4.6 Data and Baseline Methods

#### 4.6.1 Datasets

##### Seurat v4 PBMC Dataset [30]

The Seurat v4 PBMC dataset contains peripheral blood mononuclear cells (PBMCs) profiled using both transcriptomics and proteomics modalities. This dataset includes a total of approximately 161,764 cells, with 23,385 genes measured for transcriptomics and 224 antibodies targeting surface proteins in a single protein panel. This paired multi-omics dataset is particularly valuable for studying immune cell heterogeneity and serves as a benchmark for testing models that integrate RNA and protein data.

##### Seurat v3 CITE-seq BMMC Dataset [31]

This dataset profiles bone marrow mononuclear cells (BMMCs) using CITE-seq technology, providing paired transcriptomics and surface protein measurements. It contains approximately 33,455 cells, with 17,009 genes quantified at the transcriptomic level and 25 antibodies used for surface protein profiling. The dataset offers a unique opportunity to study hematopoiesis and immune cell function within the bone marrow microenvironment.

##### Liver CITE-seq Dataset [32]

This dataset comprises single-cell transcriptomics and paired surface proteomics profiling of human liver tissue. It includes approximately 73,580 cells, with 20,096 genes measured at the transcriptomic level and 187 antibodies targeting surface proteins in the proteomic panel. Data were generated as part of the Liver Cell Atlas project and are available from GEO under accession GSE192742 as well as via the Liver Cell Atlas portal (https://www.livercellatlas.org).

##### Human Tonsil Dataset

This dataset consists of CODEX multiplex imaging data of human tonsil tissues, processed from Maxfuse [6]. The dataset includes a panel of 46 antibodies, capturing spatial proteomics information at single-cell resolution within the tonsil microenvironment. It is invaluable for studying immune cell localization, tissue organization, and interactions within secondary lymphoid organs.

##### Metastatic Breast Cancer Dataset

This dataset, sourced from the Human Tumor Atlas Network[9], includes CODEX data from nine anatomic sites, along with detailed clinical annotations and paired single-cell RNA (scRNA) and single-nucleus RNA (snRNA) data. This dataset provides a comprehensive view of breast cancer metastasis, allowing us to study the molecular and spatial dynamics of tumor progression and the interactions within the metastatic microenvironment.

##### Protein-Protein Interaction

Protein-protein interaction network plays an important role in functional genomics, as they capture relationships and dependencies between genes. We leveraged the tissue-specific PPI from HumanBase dataset [33], the selected tissues are blood, bone marrow, liver, mammary gland and tonsil. Only the high-confidence interactions were downloaded from https://hb.flatironinstitute.org/download.

#### 4.6.2 Data Normalization

We applied a standard normalization pipeline using Scanpy[34], which includes total-count normalization per cell (library size normalization), followed by log-transformation and min-max scaling. We evaluated both highly variable genes (HVGs) and the full gene set; the final model was trained using the full gene set, which yielded the best performance (see Supplementary Table S1).

#### 4.6.3 PPI representations

After curating the tissue-specific PPI information from Humanbase, we trained the link prediction task using DNE [35], and extracted the embeddings for all the proteins in our dataset.

#### 4.6.4 Baseline

##### TotalVI

[3]is a deep generative model based on a variational autoencoder framework that jointly models RNA and protein measurements from CITE-seq data. The probabilistic modeling accounts for modality-specific noise, technical biases, and batch effects. TotalVI separates protein signals into background and foreground components for effective background correction, enabling accurate protein imputation and integrative analysis.

##### sciPenn

[4]employs recurrent neural networks (RNNs) to capture the temporal and sequential dependencies between transcriptomic and proteomic data. By integrating multiple CITE-seq datasets during training, sc-Penn enhances its predictive accuracy and robustness in surface protein imputation. This ensemble approach allows sc-Penn to generalize across diverse datasets, making it a valuable tool for predicting protein expression from RNA data.

##### MaxFuse

[6] introduces a novel strategy for aligning single-cell RNA data with spatial proteomics by leveraging weakly linked features. This method identifies correspondences between transcriptomic profiles and spatial proteomic data, enabling the integration of single-cell and spatial modalities. By facilitating cross-modal data fusion, MaxFuse aids in the spatial mapping of cellular functions and interactions within tissue contexts.

##### scTranslator

[5] scTranslator is a pioneering sequence-to-sequence model that applies language-model architectures to translate single-cell transcriptomes into proteomic profiles. It leverages both bulk RNA–protein datasets and single-cell CITE-seq experiments to pretrain its encoder–decoder framework, enabling it to predict surface and intracellular protein abundances from RNA inputs. It supports downstream analyses such as gene–protein interaction inference, pseudo-knockout simulations, and improved cell clustering.

### 4.7 Evaluation Metrics

#### Cell–Cell Cosine Similarity

For each cell i with true protein abundance vector $x_{i}^{\text{prot}}$ and predicted vector ${\hat{x}}_{i}^{\text{prot}}$, the cosine similarity is

$$\text{CosSim}\left(x_{i}^{\text{prot}},{\hat{x}}_{i}^{\text{prot}}\right)=\frac{x_{i}^{\text{prot}}⊤{\hat{x}}_{i}^{\text{prot}}}{{\left\|x_{i}^{\text{prot}}\right\|}_{2}{\left\|{\hat{x}}_{i}^{\text{prot}}\right\|}_{2}}.$$

We report the average over all test cells:

$$\bar{\text{CosSim}}=\frac{1}{\left|ℰ\right|}\sum_{i\in ℰ}\text{CosSim}\left(x_{i}^{\text{prot}},{\hat{x}}_{i}^{\text{prot}}\right).$$

#### Pearson Correlation

For each test cell i, let $x_{i}^{\text{prot}}$ be its true protein abundance vector and ${\hat{x}}_{i}^{\text{prot}}$ be its predicted vector. The Pearson correlation for cell i is

$$r_{i}=\frac{\sum_{p=1}^{P}\left(x_{i,p}-{\overset{‾}{x}}_{i}\right)\left({\overset{ˆ}{x}}_{i,p}-{\bar{\overset{ˆ}{x}}}_{i}\right)}{\sqrt{\sum_{p=1}^{P}{\left(x_{i,p}-{\overset{‾}{x}}_{i}\right)}^{2}}\sqrt{\sum_{p=1}^{P}{\left({\overset{ˆ}{x}}_{i,p}-{\bar{\overset{ˆ}{x}}}_{i}\right)}^{2}}},$$

where ${\overset{‾}{x}}_{i}=\frac{1}{P}\sum_{p=1}^{P}x_{i,p}$ and ${\bar{\overset{ˆ}{x}}}_{i}=\frac{1}{P}\sum_{p=1}^{P}{\overset{ˆ}{x}}_{i,p}$. We report the average Pearson correlation over all test cells:

$$\overset{‾}{r}=\frac{1}{\left|ℰ\right|}\sum_{i\in ℰ}r_{i}.$$

#### Spearman Correlation

For each test cell i, let $\text{rank}\left(x_{i,p}\right)$ be the rank of $x_{i,p}$ among {$x_{i,1},\ldots ,x_{i,P}$}, and $\text{rank}\left({\overset{ˆ}{x}}_{i,p}\right)$ be the rank of ${\overset{ˆ}{x}}_{i,p}$. The Spearman correlation for cell i is

$$\rho_{i}=\frac{\sum_{p=1}^{P}(\text{rank}(x_{i,p})-{\bar{\text{rank}}}_{i})(\text{rank}({\overset{ˆ}{x}}_{i,p})-{\bar{\hat{\text{rank}}}}_{i})}{\sqrt{\sum_{p=1}^{P}{\left(\text{rank}(x_{i,p})-{\bar{\text{rank}}}_{i}\right)}^{2}}\sqrt{\sum_{p=1}^{P}{\left(\text{rank}({\overset{ˆ}{x}}_{i,p})-{\bar{\hat{\text{rank}}}}_{i}\right)}^{2}}},$$

where ${\bar{\text{rank}}}_{i}=\frac{1}{P}\sum_{p=1}^{P}\text{rank}\left(x_{i,p}\right)$ and ${\bar{\hat{\text{rank}}}}_{i}=\frac{1}{P}\sum_{p=1}^{P}\text{rank}\left({\overset{ˆ}{x}}_{i,p}\right)$. We report the average Spearman correlation over all test cells:

$$\overset{‾}{\rho }=\frac{1}{\left|ℰ\right|}\sum_{i\in ℰ}\rho_{i}.$$

#### FOSCTTM (Fraction of Samples Closer Than the True Match)

Given two embedding sets {$z_{i}^{\text{rna}}$} and {$z_{j}^{\text{prot}}$} for N test cells, define the Euclidean distance (or cosine distance) d(i,j) between $z_{i}^{\text{rna}}$ and $z_{j}^{\text{prot}}$. For each cell i, let the rank of its true match (i,i) among $\{d(i,j){\}}_{j=1}^{N}$ be ${\text{rank}}_{i}$.

Then

$$\text{FOSCTTM}=\frac{1}{N}\sum_{i=1}^{N}\frac{{\text{rank}}_{i}-1}{N-1},$$

so that a perfect alignment yields FOSCTTM = 0.

#### Match Score

Let match (i) be the index j that minimizes d(i,j). The match score is the fraction of cells whose nearest neighbor in the opposite modality is the true partner:

$$\text{MatchScore}=\frac{1}{N}\sum_{i=1}^{N}1\left[\text{match}\left(i\right)=i\right].$$

### 4.8 Implementation Details

#### Train/Test Split Setting

During development, we employ a random train/test split to allow for internal validation. The same data distribution is shared between train and test, ensuring evaluation on held-out cells without distribution shift.

#### Out-of-Distribution (OOD) Setting

To assess robustness and generalizability, we hold out entire assays or cell types from training. In the translational RNA-to-protein and cell-type prediction tasks, any cell types omitted during training are used exclusively in the test set. This evaluates the model’s ability to generalize to novel, unseen distributions.

#### Zero-Shot and Few-Shot

In the zero-shot setting, no labeled test-domain data are used during training. For example, the translational model is trained on PBMC CITE-seq and tested on BMMC CITE-seq without any BMMC training data. In the few-shot setting, a small fraction of labeled test-domain samples (e.g., 0.5%–5%) are added to the training set to evaluate low-data generalization.

### 4.9 Pathway Analysis

To investigate the functional themes underlying the spatially patterned proteins, we performed gene set enrichment analysis in g:Profiler [36], using our predicted panel of 2,484 human surface proteins as the background set. Specifically, we selected the top 20 proteins with the highest Moran’s I scores—indicative of strong spatial autocorrelation—from two biopsy cohorts (metastatic breast cancer in liver and primary breast tumor). These 20 proteins were submitted to g:Profiler (https://biit.cs.ut.ee/gprofiler), querying against KEGG, Reactome, and other curated pathway databases. By specifying our protein list as the custom background, we obtained adjusted p-values (Benjamini–Hochberg FDR) for each term with a significance threshold of q<0.05.

## Supplementary Material

1

## Figures and Tables

**Figure 1:**
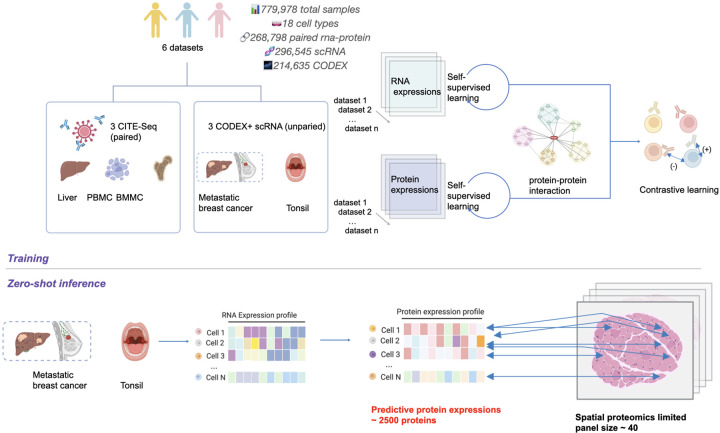
System Overview of scProSpatial. The model uses a two-stage learning strategy. Stage 1 performs self-supervised learning on RNA and protein data, guided by protein-protein interaction networks. Stage 2 applies contrastive learning to align modalities into a shared embedding space. Trained on paired CITE-seq datasets, the model enables zero-shot prediction of 2500 proteins from scRNA-seqs and maps them to spatial proteomics data with limited panels. The detailed illustration is provided in Supplemental Figure S1.

## Data Availability

The Seurat v4 PBMC dataset was extracted from the scvi-tools package (Python) and corresponds to the processed CITE-seq data described in [30]. The Seurat v3 BMMC dataset is available through GEO under accession https://www.ncbi.nlm.nih.gov/geo/query/acc.cgi?acc=GSE100866 [31]. The Liver CITE-seq dataset can be accessed via GEO at https://www.ncbi.nlm.nih.gov/geo/query/acc.cgi?acc=GSE192742 or the Liver Cell Atlas portal at https://www.livercellatlas.org [32]. The human tonsil CODEX dataset is publicly available through the Maxfuse project at http://stat.wharton.upenn.edu/~zongming/maxfuse/data.zip [6]. The metastatic breast cancer dataset from HTAN (HTAPP-MBC) is accessible through the Broad Institute Single Cell Portal at https://singlecell.broadinstitute.org/single_cell/study/SCP2702/htapp–mbc [9]. Tissue-specific protein-protein interaction networks were downloaded from HumanBase at https://hb.flatironinstitute.org/download [33].

## Code Availability

The code for scProSpatial is available at https://github.com/AdorableYoyo/scProSpatial. Scripts to reproduce the main results presented in the manuscript are also included in the repository.
