# Supplementary material for: Cross-level Cross-Scale Inference and Imputation of Single-cell Spatial Proteomics
Source: Res Sq. 2025 Jul 28:rs.3.rs-7108570. Preprint. [Version 1] doi: 10.21203/rs.3.rs-7108570/v1 (PMC12324605; doi:10.21203/rs.3.rs-7108570/v1)
Supplement: 1 [file NIHPPRS7108570V1-supplement-1.pdf]

Supplementary Material

Table S1: Ablation study on integrative pretraining configurations using masked language modeling (MLM).

Dataset Token	PPI Integration	Gene Features	MLM Loss	Relative Improvement
None (Concat)	✗	2k HVG	0.0040044	Baseline
Learnable	✗	2k HVG	0.0026244	34.4% ↓
Learnable	✓	2k HVG	0.0023850	9.1% ↓
Learnable	✗	Full Genes	0.0024276	7.5% ↓
**Learnable**	✓	**Full Genes**	**0.0021204**	**12.7% ↓**

Table S2: Few-shot ablation study showing the impact of pretraining. Metrics are averaged over five shot sizes.

Dataset	Pretraining	Cosine Similarity	Pearson	Spearman
	w/ pretraining	0.969 ± 0.003	0.897 ± 0.011	0.855 ± 0.012
PBMC	w/o pretraining	0.967 ± 0.008	0.889 ± 0.025	0.850 ± 0.023
	Improvement	**+0.21%**	**+0.90%**	**+0.59%**
	w/ pretraining	0.861 ± 0.015	0.800 ± 0.022	0.686 ± 0.022
Liver	w/o pretraining	0.839 ± 0.078	0.763 ± 0.121	0.647 ± 0.131
	Improvement	**+2.62%**	**+4.85%**	**+6.03%**
	w/ pretraining	0.963 ± 0.013	0.874 ± 0.048	0.861 ± 0.037
BMMC	w/o pretraining	0.946 ± 0.023	0.807 ± 0.089	0.810 ± 0.069
	Improvement	**+1.80%**	**+8.30%**	**+6.30%**

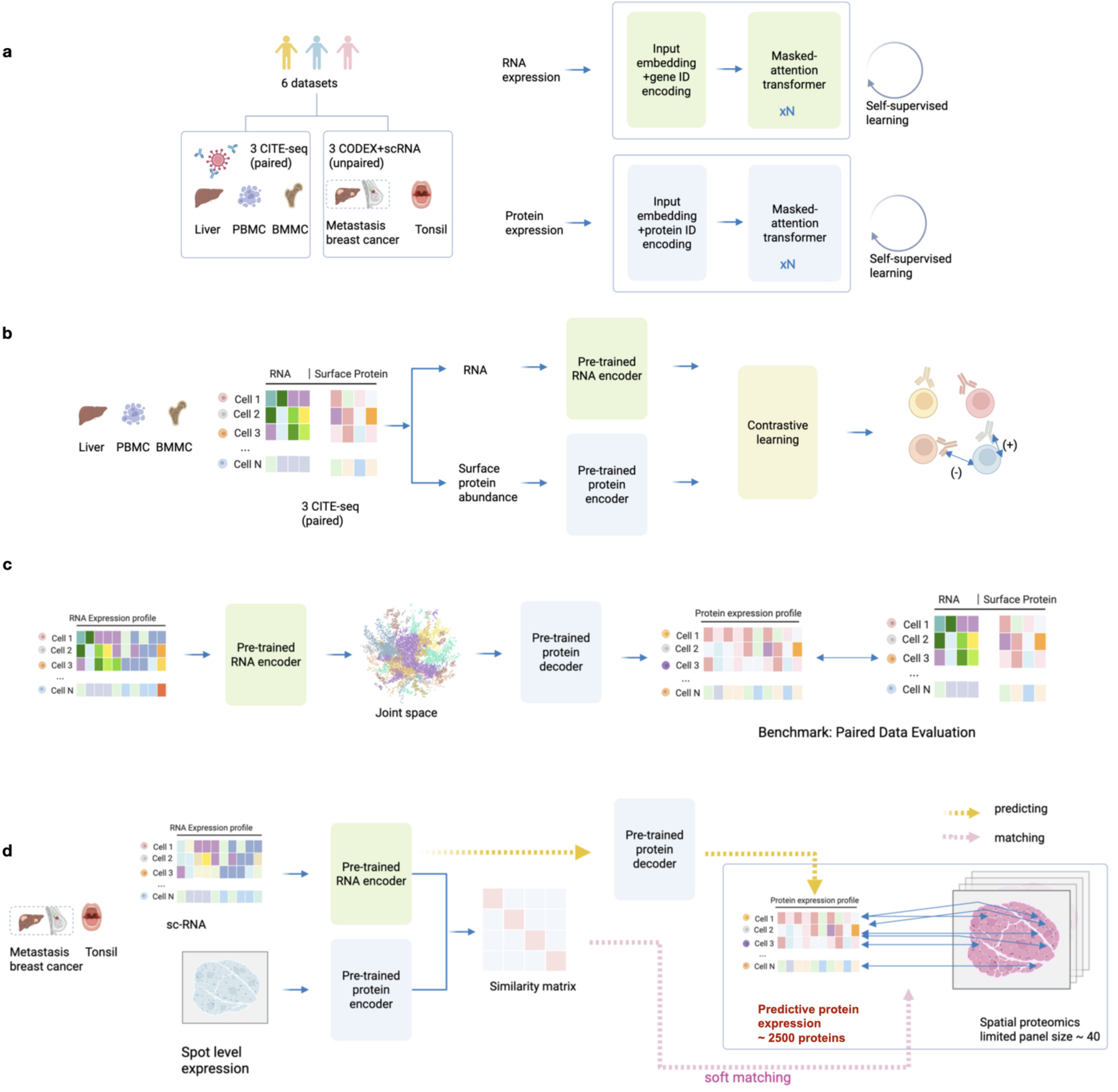

Figure S1: Detailed overview of scProSpatial framework and evaluation. a, scProSpatial is trained on both paired (CITE-seq) and unpaired (scRNA-seq and spatial proteomics) datasets using masked language modeling. b, A second-stage contrastive learning aligns RNA and protein modalities using paired CITE-seq data. c, Benchmark task: the RNA encoder predicts surface protein profiles from RNA by querying a pretrained protein decoder, evaluated under random splits, few-shot, and out-of-distribution (OOD) settings. d, Case studies: RNA profiles from tonsil and metastatic breast cancer are mapped to spatial proteomics via a shared embedding space and soft matching between scRNA-seq and CODEX.
